# Editorial: Nutrition and sleep medicine

**DOI:** 10.3389/fnut.2022.1104427

**Published:** 2022-12-13

**Authors:** Justyna Godos, Raffaele Ferri, Giuseppe Grosso

**Affiliations:** ^1^Department of Biomedical and Biotechnological Sciences, University of Catania, Catania, Italy; ^2^Sleep Research Centre, Department of Neurology I.C., Oasi Research Institute - IRCCS, Troina, Italy

**Keywords:** nutrition, diet, sleep, sleep disorders, brain health

Over the last decades, the prevalence of sleep disorders has been reported to have substantially increased globally (1). A variety of behavioral risk factors acting at an epidemiological level have been identified to explain, at least in part, this observed rise. The modern lifestyle is characterized by a number of potential contributors to poor sleep quality, such as higher levels of stress, prolonged working hours, and higher exposure to artificial lights (2); moreover, meal frugality and preference for low-quality ready-meals have also been hypothesized to play a role in sleep disorders (3). The transition from traditional dietary patterns characterized by a preference for plant-based foods toward a Western-like diet characterized by a high intake of nutrient-poor energy-dense ultra-processed food has been identified as a possible contributor (4). The relation between diet and sleep features is rather complex, being most likely bidirectional (nutritional factors affecting sleep quality and sleeping patterns affecting calorie intake, food quality, and obesity risk) (5) and multidimensional, involving other lifestyle aspects (i.e., level of physical activity, smoking and alcohol drinking habits, etc.) and health factors (i.e., presence of obesity and diabetes, sleep apnea, etc.) (6). Besides immunitary and hormonal activities, also alterations of the gut microbiota and the circadian rhythm are studied to disentangle the mechanisms underlying the relation between diet and sleep (7). Research on this topic is thus multidisciplinary, involving social and epidemiological relations between diet and sleeping patterns, or preclinical and mechanistic studies to understand which specific components of the diet may play a role in sleep features. What seems clear from the advances in the evidence produced is that both eating and sleeping represent two physiological processes that are more related than thought in the past, not just affecting “similar” pathways but rather being part of a unique larger mechanism necessary to the proper functioning of the human body.

In this Research Topic entitled “*Nutrition and sleep medicine*” various research groups provided evidence of the relation between dietary factors and sleep features from observational studies, further explored by additional studies on genetic biomarkers, while a mechanistic overview of the scientific literature contributed to summarize and better understand the retrieved associations. In the study of Sutanto et al., the authors aimed to investigate the relationship between protein intake and sleep quality in 104 healthy subjects between the age of 50 and 75 years old; the results showed that sleep duration was positively associated with dietary tryptophan to large neutral amino acid ratio, with significant results specific to plant tryptophan. Other two studies investigated dietary parameters and sleep quality in school children; the study of Shih et al. analyzed data from the Nutrition and Health Surveys in Taiwan involving 2,628 participants showing that those consuming more high-sugar sweetened beverages exhibited shorter sleep durations on school days and >2 h of sleep debt than those reporting low intake; the study from Ramirez-Contreras et al. was instead conducted on 588 children aged 5–12 years revealing an association of regular fish and vegetable consumption with the advance of the midpoint of sleep and fish and daily fruit consumption with fewer sleep disturbances, while the daily consumption of sweets and candy and having pasta or rice >5 times/week were significantly associated with a decrease in sleep duration. Two studies used genetic biomarkers from the UK Biobank to investigate the relation between dietary factors and sleep outcomes: the study of Zou et al. showed that genetically predicted short sleep duration is a potential causal risk factor for hyperuricemia for women but has little effect onmen; the study of Tang et al. reported that genetically increased triglyceride levels have independent causal effects on risk of sleep apnea. Finally, the study of Benton et al. summarized the evidence from the scientific literature on the role of carbohydrates on sleep features, emphasizing two major hypotheses for their potential benefits, including the increase in the uptake of tryptophan by the brain, where it is metabolized into serotonin and melatonin (hence resulting in improved sleep), but also the emerging role of glucose-sensing neurons associated with the sleep-wake cycle in the hypothalamus, which may be affected by the carbohydrate-induced changes in the level of blood glucose.

In conclusion, this Research Topic provided an interesting update of current evidence on the relationship between diet and sleep features. Nonetheless, the topic investigated needs further attention in future research while more preclinical studies are necessary to understand the mechanisms underlying the observed relation.

## Author contributions

All authors listed have made a substantial, direct, and intellectual contribution to the work and approved it for publication.
